# Attitudes and Experiences of Community Palliative Care Nurses Regarding Pediatric Home-Based End-of-Life Care: A Statewide Survey

**DOI:** 10.1177/08258597241284286

**Published:** 2024-09-26

**Authors:** Mia Helyar, Marisa Eamens, Sandra Coombs, Therese Smeal, Martha Mherekumombe, Tiina Jaaniste

**Affiliations:** 1 Department of Palliative Care, 63623 Sydney Children's Hospital, Randwick, Australia; 2 School of Clinical Medicine, 98994 University of New South Wales, Kensington, Australia; 3 Department of Palliative Care, 8538 Children's Hospital at Westmead, Westmead, Australia; 4 Palliative Care Service, 1511 South Western Sydney Local Health District, Liverpool, Australia

**Keywords:** community, home, end-of-life, nurse, palliative, pediatric

## Abstract

**Objectives:** Pediatric end-of-life (EOL) care at home is often provided by community palliative care (CPC) nurses who do not specialize in pediatrics. This study aimed to better understand the challenges CPC nurses face when providing EOL care to children at home. **Methods:** A total of 52 CPC nurses across New South Wales (NSW), Australia, participated in an online survey about their training, attitudes, and experiences regarding the provision of home-based pediatric EOL care. Participants were asked to reflect back over a “negative” experience of caring for a child at EOL, where things did not go as well as hoped, and a “positive” EOL care experience, where nurses perceived that care of the child and family went well, and respond to questions about these experiences. **Results:** Confidence of CPC nurses when providing EOL care to pediatric patients was significantly lower than when caring for adults (*p*'s < .05). Most respondents expressed the desire for more training in pediatric EOL care. Cases identified as negative by CPC nurses did not significantly differ from positive cases in terms of the timing of the referral to CPC, clinical symptoms at EOL, or how well informed the nurses felt. Siblings were present at EOL in 74% of the negative experiences and 86% of the positive experiences, reportedly receiving significantly poorer support in the negative experiences (*p *= .002). **Conclusion:** This research contributes to an improved understanding of the challenges associated with home-based pediatric EOL care and highlights potential areas for improvement in CPC service delivery and training.

## Introduction

Despite significant advancements in medical technology, many children still suffer from life-limiting illnesses.^1-4^ These are conditions from which there is no reasonable hope of cure and from which children will ultimately die.^1,2^ There is an increasing number of children requiring palliative care and subsequent end-of-life (EOL) care.^1,5,6^ EOL care refers to the provision of holistic care to support children and their families during the final stage of life, which can last from hours to days, and in some cases weeks before death.^7^ Pediatric EOL care can occur in various locations including home, hospital, or hospice, however, many families and healthcare providers prefer home-based EOL care for children.^8-12^

Potential benefits associated with effective pediatric EOL care in the home setting include familiarity and comfort for the patient and family,^11,13-15^ parental empowerment in taking an active role in their child's care,^11,16-18^ greater opportunity for sibling involvement,^15,19-21^ reduction in need for hospitalizations,^22^ and greater flexibility to accommodate religious and cultural practices.^8,23^ Nevertheless, home-based pediatric EOL care is not preferred by all families and is not a practical or achievable option for some.^24^

Large distances and workforce limitations make it impossible for specialist pediatric palliative care services to always provide direct care to children in their local communities.^25^ Therefore, home-based pediatric EOL care is often provided by generalist community-based nursing teams, who are supported by specialist palliative care nurses, with neither group of nurses specialized in pediatrics.^26-28^ These two groups are in turn supported by specialist pediatric palliative care services. Effective EOL care for children differs from that of adults, requiring knowledge about pediatric medications and dosages,^29,30^ knowledge of life-limiting conditions seen in childhood,^22,29,30^ and ability to provide psychosocial support to pediatric patients, caregivers, and siblings.^31^

There is limited literature on the challenges faced by the predominantly adult-focused CPC nurses when caring for children at EOL. Potential challenges that have been noted in the literature include difficult access to specialized pediatric resources and medical equipment^32^ staying within professional boundaries,^29,33,34^ the prolonged and time-consuming nature of pediatric palliative care,^35^ and the emotional toll faced by the nurse.^33,34,36-38^ It is not well documented what types of pediatric EOL symptoms CPC nurses are required to manage, nor their perceived management adequacy. Pediatric EOL symptoms have previously been documented in hospital-based contexts,^39^ or not differentiating between hospital or home-based contexts.^40^ Some pediatric symptom data is available for the home-based EOL context,^41^ this being based on parental recollection 6-36 months after the death.

To date, the available research exploring home-based pediatric EOL care has primarily been from the perspective of caregivers.^42,43^ Some qualitative data has been reported with nurses providing home-based pediatric EOL care,^34,35^ but little quantitative data is available. The over-arching aim of this study was to identify challenges faced by CPC nurses when caring for a child at home at EOL. Specific aims were: (1) To assess the amount of past training, experience, and confidence among CPC nurses for caring for patients of various ages. (2) To assess the timeliness of community palliative care referrals for pediatric patients. (3) To assess whether the amount of information received by CPC nurses differed for pediatric EOL cases that went well (“positive” cases) relative to those that were not perceived as going well (“negative” cases). (4) To identify the frequency with which CPC nurses reported that the home environment was not conducive for EOL care and the nature of challenges encountered. (5) To document the frequency of physical symptoms that pediatric patients experienced during EOL at home (in both negative and positive experiences) and the adequacy of symptom management provided, as perceived by CPC nurses. Gaining a better understanding of the CPC nurses’ perspectives and experiences is essential to better inform the development of service delivery models and programs to enhance EOL care and support at home for children and their families.

## Methods

The research reported in this manuscript is the first phase of a two-phase explanatory sequential mixed-methods design.^44^ This first phase consisted of a predominantly quantitative survey. The second qualitative phase of the project consisted of a semistructured interview, with a subset of participants from the current study, and will be reported elsewhere. This project was approved by the Sydney Children's Hospitals Network Human Research Ethics Committee (2022/ETH02393).

### Participants

Participants were CPC nurses recruited from metropolitan, regional, and rural locations of New South Wales (NSW), Australia. Invitations were emailed to CPC nurses working throughout NSW. Recruitment also occurred through email distribution lists of the NSW Palliative Care and End-of-Life Service Development Officers, and an online news digest sent out by the Agency for Clinical Innovation End-of-Life and Palliative Care Network.

Study inclusion criteria required that participants: (1) were employed as specialist CPC nurses, (2) worked for a service that accepts pediatric palliative referrals, and (3) had cared for at least one child at EOL in the last 5 years. A total of 62 nurses agreed to participate; however, 10 did not meet the full eligibility criteria. Thus, 52 nurses completed at least some of the online survey.

### Measures

The survey was developed for the purposes of the current study and piloted for clarity and relevance by an experienced CPC nurse. This individual did not subsequently participate in the study. The survey covered the following areas: (1) Nurse demographics. (2) Amount and type of EOL training received and confidence levels in providing EOL care to patients of different ages. (3) Nurses were asked to identify a pediatric EOL care experience they had been involved in that they felt had not gone well (“negative” case) and an experience they felt had gone well (“positive” cases). With a negative and then a positive patient care experience in mind, respondents were asked about the timeliness of community referral, amount of patient-related clinical and psychosocial information received at referral, perceived suitability of the home environment for EOL care provision, who was present at the time of EOL care, and physical symptoms experienced by the child at EOL.

A variety of well-validated response options were used in the survey, including 0-10 numeric rating scales with clear verbal anchors (eg “not at all well,” “extremely well”), checklist-style responses, and yes/no/unsure responses.

### Procedure

Potential participants were emailed the study participant information sheet and link to an online consent form via the RedCAP^45^ platform. Upon providing consent, participants who met eligibility criteria could proceed with the survey, which took approximately 20-30 minutes to complete. Nurses who completed the consent form but not the survey were sent a reminder email two weeks later. Respondents were asked to rate their distress levels at survey completion. If distress was rated as more than 6/10, a member of the research team would call them within 48 hours.

### Statistical Analyses

Data was analyzed using IBM^®^ SPSS^®^ Statistics 27. Descriptive analyses were conducted. Variables were screened for normality (skewness <1 and kurtosis <3). If normality assumptions were not met, then nonparametric tests were utilized. Parametric (*t*-tests) and nonparametric (Mann-Whitney U tests) tests were used to compare various responses for positive versus negative experiences. Chi-square tests were used to assess for associations with categorical data.

## Results

### Demographic Information

A total of 52 CPC nurses participated in the online survey. The demographic characteristics of CPC nurses are shown in Table 1. The length of time working in CPC ranged from 2-32 years (*M* = 13.23 years, *SD* = 7.64).

**Table 1.** Demographic characteristics of CPC nurses with experience caring for a pediatric patient (*n* = 52).

Nurse demographics	*n*	%
Gender		
Female	49	94.2
Male	3	5.8
Age		
30-39	4	10
40-49	5	12.5
50-59	18	45
60-69	12	30
Prefer not to say	1	2.5
Geographical location		
Metropolitan	19	40.4
Regional	8	17
Rural	13	27.7
Regional and Rural	7	14.9

*Note*. Sample sizes for individual variables may be lower than the complete sample size due to missing data. Percentages are derived from the total number of responses for each respective question*.*

### Experience, Training, and Confidence in Pediatric EOL Care Among CPC Nurses

Most CPC nurses completing the survey had not cared for a neonate (85.7%), infant (97.1%), child (68.6%), or adolescent (65.7%) at EOL within the past 12 months, while many had cared for over 20 adult patients (80%) at EOL. Furthermore, 49% of CPC nurses reported having “hardly any” pediatric EOL training, while only 5.9% reported the same for adult EOL training. Most CPC nurses identified the need for more training across all age groups (neonate: 82.4%, infant: 84.3%, child: 96.1%, adolescent: 94.1%, and adult: 72.5%). Self-reported confidence in providing EOL care was lowest for neonates, increasing with each successive age group (see Table 2). Confidence to provide EOL care to adult patients was significantly higher than in each of the pediatric age groups (all *p*'s < .05).

**Table 2.** CPC Nurse Confidence Rating (0-10) in Providing EOL Care at Home to Patients of Different Ages.

Age category	Median	IQR	Range
Neonate	2	4	0-10
Infant	3	4.75	0-10
Child	5	3	0-10
Adolescent	6	3.75	0-10
Adult	10	3.75	1-10

*Note.* Age categories are defined as follows; neonate (<4 weeks), infant (4 weeks-1 year), child

(2-12 years), adolescent (13-17 years) and adults (18+).

### Negative Experiences of CPC Nurses

When rating their negative EOL care experiences on several outcome indicators, the poorest outcome was the nurse's own psychological well-being in response to the experience (*M* = 5.96, *SD* = 2.5). The best rating was for achieving a meaningful and/or peaceful environment (md = 8, IQR = 4). There was much variability in each of the outcomes (see Table 3).

**Table 3.** How Well Outcomes Were Achieved in the Negative Experience.

	Mean	*SD*	Median	IQR	Range
Management of patient's symptoms	6.18	2.25	6	3	1-10
Family's cultural, spiritual, and or religious needs met	6.20	2.29	6	3	1-10
Adequate support for family members	6.00	2.67	6	4	0-10
Meaningful and/or peaceful environment *	7.04	2.40	8	4	0-10
Smooth post-death arrangements *	6.22	3.06	7	4	0-10
Your satisfaction with your role performed *	6.37	2.32	7	2	0-10
Your psychological well-being	5.96	2.50	6	3.5	1-10

*These variables were nonparametric.

When asked to identify potential areas for improvement in the provision of EOL care in those negative experiences, 65.3% of CPC nurses expressed the need for more pediatric EOL experience. Over half (53.1%) highlighted the need for education and training related to psychosocial management (see Table 4).

**Table 4.** Potential Areas for Improvements in Negative EOL Care Experiences Identified by CPC Nurses.

Potential improvements	*n*	%
*More experience/education:*		
More experience needed with pediatric patients at EOL.	32	65.3
Education on psychosocial management of family	26	53.1
Education on communication skills in pediatric EOL care	22	44.9
Education on clinical/symptom management	19	38.8
*Information needs*		
More information about patient's condition	10	20.4
More information about family's cultural, religious, and/or spiritual practices.	8	16.3
*Resources/Supports*		
Would have benefit/ed from meeting family earlier to establish more rapport.	16	32.7
Limited or lacking resources (eg medications, equipment)	10	20.4
Needed additional health personnel to help manage patient's/family's needs.	11	22.4
Didn’t know who to ask for advice when needed	4	8.2
*Other*	6	12.2

### Timing of Referrals

Many pediatric patients requiring community-based EOL care were referred to the CPC team *before* needing actual EOL care (83.7% for negative patient experiences and 84.2% for positive patient experiences). However, 16% of patients were not referred to CPC until EOL care was required. There was no significant difference between the negative (median = 10 weeks, IQR = 18) and positive (median = 10 weeks, IQR = 11) experiences in terms of time between referral to the CPC team and death (*U *= 570.5, *p *= .69).

### Information Received by CPC Nurses

When reporting how well-informed CPC nurses felt in 5 key areas relevant to EOL care provision, there were no significant differences between negative and positive experiences (all *p*'s > .05; see Table 5). The median response for how well-informed CPC nurses felt was 7 or 8 out of 10 for all types of information in both positive and negative experiences. However, there was wide variability in the amount of information received by CPC nurses, with some nurses reporting having received little or no information about key areas relevant to EOL care provision.

**Table 5.** How Well-Informed Nurses Felt in key Areas Relevant to Patient and Family Care (0 = Poorly Informed, 10 = Extremely Well Informed).

Type of information	Negative experience			Positive experience			Significance (Mann-Whitney)	
	Median	IQR	Range	Median	IQR	Range	*U*	*p*
Patient's condition	8	3	3-10	8	1.75	4-10	1032.5	.171
Interventions/comfort measures	8	4	2-10	8	2	3-10	1002.5	.276
Family dynamics	8	3.5	0-10	8	3	4-10	934.0	.638
Cultural/spiritual	8	4	1-10	8	3.75	3-10	988.0	.338
How different healthcare professionals will be involved	7	4	2-10	8	2	3-10	1009.5	.250

### Home Environment

As shown in Table 6, it was common for siblings to be present during EOL care (in 73.5% of negative experiences and in 86% of positive experiences). Nurses perceived that siblings received significantly less support in the negative experiences (median = 6, IQR = 4) relative to the positive experiences (median = 8, IQR = 3.75; *U *= 815.5, *p *= .013). Post hoc analyses revealed that CPC nurses rated that 42.1% of siblings in the negative experiences had support ratings of less than 5/10 (0 = not at all well supported, 10 = extremely well supported).

**Table 6.** Individuals Present for Pediatric EOL Care in the Home Environment & Perceptions by CPC Nurse of Levels of Support During EOL Care.

Present at EOL								
	Negative experiences (*N* = 49)^a^				Positive experiences (*N* = 37)^a^			
	*n*		%		*n*		%	
Parents	49		100		37		100	
Siblings	36^b^		73.5		32^b^		86	
Extended family	30		61		24		65	
Friends	17		35		16		43	
NDIS^c^ carer	4		8		2		5	
	Perceptions of support received (0-10) (0= “not at all well supported,” 10= “extremely well supported”)							
Level of support for this person:	Negative experience			Positive experience			Significance (Mann-Whitney)	
	Median	IQR	Range	Median	IQR	Range	*U*	*p*
Primary Caregiver	7	3	3-9	8	2	4-10	1040.5	.090
Second Caregiver	6	4	4-10	8	3	4-9	598.5	.380
Sibling(s)	6	4	0-10	8	3.75	3-10	815.5	.013
Nurse	7	4	0-10	8	2	3-10	1073.0	.086

a Sample sizes for the negative and positive experiences are different due to more missing data for the positive experiences. Percentages are derived from the total number of responses for each respective question.

b In the negative experience, 5 patients did not have siblings and in the positive experience, 2 patients did not have siblings

c NDIS = National Disability Insurance Scheme

There were on average 3.7 (*SD *= 1.76) different health professional disciplines present for the provision of EOL care in the negative experiences and 4 (*SD *= 1.86) different health professional disciplines present in the positive experiences, with this difference not being statistically significant (*t *= .81, *p *= .37).

When CPC nurses were asked about how conducive the home environment was for EOL care provision, in their negative experiences they reported that 26.5% (*n* = 13) of home environments were less than optimal for EOL care provision. In the positive experiences, there were 13.5% (*n* = 5) home environments that were considered less than optimal. Table 7 shows reasons endorsed by nurses for why some home environments were less than optimal for EOL care.

**Table 7.** Reasons Given for why Some Home Environments Were Considered by CPC Nurses to be Nonconducive for EOL Care.

	Negative experience (*n* = 13)		Positive experience (*n* = 5)	
	*n*	%	*n*	%
Inadequate/ small space	6	46.2	3	60
Poor hygiene	2	15.4	4	80
Other children pose competing demands in a way that distracts from care provision	4	30.8	2	40
Competing demands by other people (not children) within the household which distracts from care provision	4	30.8	3	60
Other reason	3	23.1	1	20

*Note.* Significance testing was not conducted due to small sample sizes

### Presence of EOL Symptoms and Management

CPC nurses reported that most pediatric patients experienced at least some symptoms during EOL (93.8% in negative experiences and 91.7% in positive experiences; see Table 8). The most common patient symptoms included pain and agitation. In the negative cases, nurses perceived that they managed pain and agitation significantly more poorly than in the positive cases.

**Table 8.** Symptom Occurrence and Management Adequacy at EOL as Perceived by CPC Nurses.

	Symptom occurrence		Perceived management adequacy (0-10*)		
	Negative cases (*n* = 49)	Positive cases (*n* = 36)	Negative cases *M* (*SD*)	Positive cases *M* (*SD*)	Significance # (*p*)
Pain	30 (61%)	25 (69%)	6.20 (2.07)	7.72 (1.57)	.004
Agitation	26 (53%)	20 (56%)	5.54 (2.44)	7.70 (1.66)	.001
Breathlessness	19 (39%)	10 (28%)	5.74 (2.13)	7.20 (1.14)	.054
Secretions	16 (33%)	11 (31%)	6.00 (2.28)	7.18 (2.14)	.187
Seizures	14 (29%)	12 (33%)	7.71 (1.86)	8.08 (1.38)	.576
Nausea/vomiting	5	4	6.25 (0.96)	8.00 (1.41)	–
Constipation/diarrhea	4	3	7.00 (2.55)	5.67 (2.89)	–
Other	1	0	2.00 (-)	–	–

*0 = not at all well, 10 = extremely well.

# Significance testing was not carried out where there were 5 or fewer occurrences of a symptom.

A total of 63.3% of CPC nurses reported that in their negative experiences, optimal patient care was still achieved. When reporting positive experiences, this percentage was significantly higher (80.6%; *χ*² = 8.017, *p *= .005).

## Discussion

This study contributes to the understanding of home-based pediatric EOL care, particularly from the perspective of nonpediatric specialist CPC nurses. The study has shed light on challenges faced by CPC nurses in striving to achieve optimal home-based management for pediatric patients and their families. Most CPC nurses completing the survey had not cared for a pediatric patient at EOL in the past 12 months. About half reported having “hardly any” pediatric EOL training. Consequently, it is not surprising that CPC nurse confidence levels were significantly lower for all pediatric age categories relative to adult patients requiring EOL care. Furthermore, confidence levels were successively lower for each younger age category. These findings are in line with other literature documenting that many CPC nurses do not feel confident^32,46^ or lack adequate pediatric training^32,35,47-49^ in providing home-based pediatric EOL care.

Sixteen percent of patients were not known to the CPC team before requiring EOL care. Although considerable attention has been given to promoting early referrals to pediatric palliative care teams,^50-52^ more attention needs to be directed to the timing of referrals to CPC teams. Earlier referrals to CPC teams enable greater opportunity for CPC nurses to get to know the family and establish trust,^35^ and to assess the home environment, suggesting equipment or adaptations where needed, before providing EOL care. Late referrals make it harder for CPC nurses to plan ahead for optimal outcomes; instead adopting a reactive approach to circumstances as they arise.

The current study examined how well-informed CPC nurses felt, based on information provided to them, across various areas relevant to pediatric EOL care provision. There was much variability in the amount of information that CPC nurses reported receiving, with some nurses reporting having received little or no relevant information on key issues relevant to patient care. However, there was no significant difference in the amount of information received for negative cases relative to positive cases, suggesting that CPC nurses were able to work around challenges associated with limited information. Nevertheless, routine, comprehensive, and timely information provision to CPC teams is of key importance in striving for seamless transitions between hospital and home, and optimal care coordination.^53^

When considering the negative pediatric EOL home-based care experiences, CPC nurses reported that about one-quarter of patient homes were less than optimal in some respect for EOL care. Perceived challenges identified were most commonly related to inadequate space, competing demands from other children or adults, or hygiene. This may be of particular concern in the context of late referrals to CPC teams when there is less opportunity to make plans for managing environmental challenges. Consequently, nurses may feel less prepared or in control of the situation.^35^ To our knowledge the current study provides the first published data regarding challenging aspects of the home environment in relation to home-based pediatric EOL care provision.

It was identified that siblings were commonly present for the EOL care of their unwell brother or sister in the home setting. However, CPC nurses reported that they did not feel that siblings were always well supported, especially in the negative EOL care experiences. In complex situations, where there may be particular challenges in managing the EOL care of the patient or other psychosocial complexities in the family system, the needs of well-siblings may get overlooked. It is not known how this further impacts or potentially compounds the known long-term impacts of losing a sibling in childhood or adolescence.^54,55^

CPC nurses reported that most pediatric patients receiving home-based EOL care experienced some physical symptoms, with pain and agitation the most common symptoms. This aligns with other published data regarding symptoms experienced in the context of hospital-based EOL care.^39^ The current data showed that CPC nurses reported that these symptoms were managed more poorly in the negative experiences relative to the positive experiences.

### Limitations

Nurses with a particular interest in pediatrics, or those who recalled challenging pediatric EOL care experiences, may have been more likely to participate in this research. In light of the broad methods of advertising the study through various communication platforms, it is not possible to know how many CPC nurses received the study invitation and therefore what the participation rate was. Given the infrequency of pediatric EOL care situations, it may have been difficult for nurses to identify or recall distinctly negative or positive experiences. These experiences might have occurred a considerable time ago, thus potentially introducing recall bias.^56^ For nurses with more experiences to choose from, it is not known how typical these experiences were for them.

There was more missing data for questions about positive cases. Nurses may have been more motivated to share information about their negative EOL care experiences, which may have been more salient and memorable. Questions about negative experiences were also earlier in the survey, so it is possible that participants did not complete the questions about positive experiences due to being interrupted before survey completion.

Finally, the EOL care experiences were reported from the perspective of CPC nurses. It is not known how these perspectives might differ from those of families.

### Future Research and Clinical Directions

The results of the current study flag potential value in interventions that enhance pediatric EOL care training, support, and interagency collaboration.^57,58^ Evidence-based development and subsequent evaluation of such interventions is warranted. For example, evaluating the impact of tailored educational programs on nurses’ confidence in administering pediatric medications would allow for actionable insights to improve practice. Although there is a growing body of research investigating the needs of well-siblings,^59-63^ future research could address the nature of well-sibling needs and support specifically in the home-based EOL care context.

Nurses who may be required to care for children at EOL would benefit from increased exposure to pediatric palliative care through supported work experience and supervised clinical placements in pediatric services. Additionally, more professional development opportunities are needed with a pediatric EOL care focus, both as part of routine professional development and at the time of pediatric patient referral. Further investment is warranted in the increased use of the pop-up model of pediatric palliative care,^64-66^ involving access to in-time training and specialist pediatric palliative care support to local teams. Further CPC training should encompass a range of skills, including managing psychosocial factors and promoting nurses’ psychological well-being. Utilizing existing online pediatric-specific palliative care modules^67,68^ or developing online tailored pediatric EOL home-care modules may serve as valuable learning tools for CPC nurses. Increased specialist pediatric outreach services, especially those offering in-person support, may be beneficial in supporting CPC nurses who are less familiar with pediatric EOL care provision. Moreover, it would be valuable to allocate additional funds within specialist pediatric palliative care services to encourage initiatives such as tele-education sessions supporting CPC nurses^69^ and more frequent joint home visits involving specialist pediatric palliative care nurses, mentorship programs,^35^ and formal prebrief^70,71^ and debrief^72,73^ sessions. These prebrief and debrief sessions may allow CPC nurses to address psychosocial concerns, access support, and share knowledge across the team.^73,74^

## Conclusion

This study explored the complex nature of pediatric EOL care, focusing on the challenges experienced by CPC nurses providing home-based EOL care to children within what is predominantly an adult-based healthcare framework. Patient clinical symptoms and timing of the referral to CPC were not found to differ between the positive versus negative experiences reported by CPC nurses. However, psychosocial factors, such as the adequacy of support for siblings, were found to be significantly poorer for negative cases. There was much variability in how well-informed CPC nurses felt with respect to various aspects of referral information. The results of the study highlight the need for improvements in interagency collaboration to ensure adequate information sharing, as well as additional education and training for pediatric EOL care. Future clinical and strategic directions should focus on building capacity and confidence in CPC teams providing EOL care to pediatric patients in their homes.
